# How well do postpartum blood loss and common definitions of postpartum hemorrhage correlate with postpartum anemia and fall in hemoglobin?

**DOI:** 10.1371/journal.pone.0221216

**Published:** 2019-08-22

**Authors:** Holly Anger, Jill Durocher, Rasha Dabash, Beverly Winikoff

**Affiliations:** Gynuity Health Projects, New York, NY, United States of America; Centre Hospitalier Departementai Vendee, FRANCE

## Abstract

**Objective:**

We aimed to better understand how well postpartum blood loss and common postpartum hemorrhage (PPH) definitions (i.e. blood loss ≥500ml = PPH, ≥1000ml = “severe” PPH) correlate with postpartum anemia and fall in hemoglobin.

**Methods:**

Secondary analysis of data from three randomized trials that objectively measured postpartum blood loss and pre- and post-delivery hemoglobin among vaginal deliveries: one trial included 1056 home-births in Pakistan and two multi-country hospital-based trials included 1279 women diagnosed with PPH. We calculated Spearman’s correlation coefficients (r_s_) for blood loss with hemoglobin drop and postpartum hemoglobin, and we compared PPH blood loss markers (≥500ml, ≥1000ml) with large hemoglobin drops (≥2 g/dL) and the threshold for moderate postpartum anemia (<10g/dL).

**Results:**

In the Pakistan study and the multi-country trials, blood loss was weakly correlated with hemoglobin drop (Pakistan: r_s_ = -0.220, multi-country trials: r_s_ = -0.271) and postpartum hemoglobin (Pakistan: r_s_ = -0.220, multi-country trials: r_s_ = -0.316). In both the Pakistan and multi-country trials, hemoglobin drop ≥2 g/dL occurred in less than half of women with 500–999 ml blood loss (55/175 [31%] and 302/725 [42%], respectively) and was more common among women who bled ≥1000ml (19/28 [68%] and 347/554 [63%], respectively). Similarly, in the Pakistan and multi-country trials, postpartum anemia <10 g/dL was less frequent among women who bled 500–999 ml (55/175 [31%] and 390/725 [54%], respectively) and more frequent among women with ≥1000ml blood loss (20/28 [71%] and 416/554 [75%], respectively).

**Conclusions:**

Postpartum morbidity as measured by hemoglobin markers was common for women with blood loss ≥1000ml and relatively infrequent among women with blood loss 500-999ml. These findings reinforce the importance of severe PPH as the preferred outcome to be used in research. The weak correlation between blood loss and hemoglobin markers also suggests that this relationship is not straightforward and should be carefully interpreted.

## Introduction

Though blood loss in the immediate postpartum period is expected for all women, an excessive amount (i.e. postpartum hemorrhage [PPH]) can have serious consequences and is the leading cause of maternal mortality globally [1]. The question of what constitutes excessive blood loss is one of continued debate. The definition specified by the World Health Organization (WHO) is the most frequently cited, defining PPH as ≥500 ml blood loss within 24 hours of a vaginal birth and ≥1000 ml blood loss following a cesarean birth [2, 3]. Consensus around this definition was formed in the 1950s and 1960s, when several articles provided rationale for the 500 ml threshold [4–7]. However, there is little published evidence showing that this threshold is an indicator for PPH-related morbidity [8] and evidence from studies that treated women at later thresholds of bleeding (e.g. 700 ml) suggests that many women who bleed 500 ml may be fine without intervention [9, 10]. Notably, the American College of Obstetricians and Gynecologists (ACOG) updated their definition of PPH to be “blood loss greater than or equal to 1000 ml or blood loss accompanied by signs or symptoms of hypovolemia”[11]. Nevertheless, in many settings throughout the globe, the 500 ml definition continues to be a guiding norm that is used in clinical practice guidelines, program evaluation approaches and research studies.

Postpartum hemoglobin alone or as compared to pre-delivery levels can help gauge the physiological impact of delivery and postpartum blood loss. Postpartum blood loss and change in pre- to post-delivery hemoglobin are presumed to be highly correlated; however, several studies report poor correlation between the two [12–14]. Recent studies show the two to be significantly correlated only when postpartum blood loss exceeds 1500 ml [15, 16], which may suggest that the exact amount of blood loss below this amount may play a small role in impacting a woman’s physiological well-being after delivery. Women may tolerate smaller amounts of blood loss (500–1000 ml) with few complications; thus, the 500 ml threshold may be a poor indicator of a negative outcome [17]. The implications of this are especially relevant for maternal health research purposes, where exact postpartum blood loss and the 500 ml PPH threshold are commonly used outcomes, even though they may hold relatively little clinical significance. Analyzing the relationship of blood loss and blood loss-based definitions of PPH with postpartum hemoglobin outcomes may help shed light on this issue, as postpartum anemia can be a consequence of a clinically important loss of blood during delivery that has negative consequences for maternal and child health in the postpartum period [18–20]. In effort to better understand how well postpartum blood loss and common postpartum hemorrhage (PPH) definitions (i.e. blood loss ≥500 ml = PPH, ≥1000 ml = “severe” PPH) correlate with postpartum anemia and fall in hemoglobin, we conducted a secondary analysis of data obtained from three studies that systematically recorded blood loss and hemoglobin outcomes in diverse delivery populations.

## Materials and methods

We performed a secondary analysis of data collected from 3 separate randomized, placebo-controlled trials to compare measured blood loss among vaginal births with both change in pre- to post-delivery hemoglobin and postpartum hemoglobin. We hypothesized that postpartum morbidity as measured by fall in hemoglobin and by postpartum anemia would be relatively rare among women with blood loss that exceeded the commonly used PPH threshold of 500ml and did not exceed threshold of “severe” PPH (≥1000ml). The trial data included in this analysis were chosen because they are derived from some of the few trials that conducted systematic, objective assessment of blood loss and pre- and post-delivery hemoglobin, which was measured using a Hemocue handheld device (Hemocue, Angelholm, Sweden).[9, 10, 21] These data represent a convenience sample that was readily available to the authors, and though the related studies are not the only trials that have measured the parameters of blood loss and hemoglobin outcomes, other published trials have not included a direct, individual-level comparison of blood loss and hemoglobin measures and thus data presented in those publications could not be used for this analysis. In addition, these trials reflect diverse study populations from both hospital- and community-based settings, which aids in the generalizability of the findings. These published trials had the primary aim to assess safety and efficacy of uterotonics in the prevention and treatment of PPH and are registered with clinicaltrials.gov (#NCT00120237, #NCT00116350).

The first trial included home-births attended by traditional birth attendants in rural Pakistan to compare the use of 600 mcg misoprostol to placebo administered during the third stage of labor for prevention of PPH [21]. Pre-delivery hemoglobin was measured during a third trimester antenatal care visit. Postpartum blood loss was measured by placing a bedpan and perineal sheet under the woman after delivery of the baby and then pouring collected blood into a jug. Blood loss was later calculated by weighing the jug, sheet and cotton roll used to absorb blood during delivery and subtracting the known dry weight of the jug, sheet and cotton. Local study staff later visited women 3–5 days after delivery to measure the post-delivery hemoglobin.

The remaining two trials were conducted at secondary and tertiary level hospitals to compare the use of 800 mcg sublingual misoprostol and 40 IU IV oxytocin for treatment of postpartum hemorrhage under two distinct clinical scenarios: one where women received routine prophylactic oxytocin during the third stage of labor [10] and the other where women had not received prophylactic oxytocin [9]. These two independently powered studies were conducted in nine facilities in five countries (in Burkina Faso, Egypt, Turkey and Vietnam for the study among women who received prophylactic oxytocin, and in Egypt, Ecuador and Vietnam for the study among women who did not receive prophylactic oxytocin). Pre-delivery hemoglobin was measured during early labor and postpartum blood loss was measured using a calibrated polyurethane blood collection drape (Brasss-V Drapes, Excellent Fixable Drapes, India) placed under the woman immediately after delivery of the baby. The collection drape was left in place for a minimum of one hour after delivery. If active bleeding was still present after one hour, the drape was left in place until active bleeding stopped. Need for treatment for PPH was determined by clinical judgement or by measured blood loss ≥700 ml, whichever occurred first. Post-delivery hemoglobin was only assessed for women who received treatment for PPH (using the criteria described above) and was measured at least 12 hours after removal of intravenous lines or just before discharge if discharge occurred before 12 hours.

The original research was conducted in accord with Good Clinical Practices for human subjects research. The protocol for the Pakistan prevention study was approved by the Ethical Review Committee at the Aga Khan University (Karachi, Pakistan) and the protocol for the multisite treatment studies was approved by the Western Institutional Review Board (Seattle, WA, USA) and relevant institutional review boards in participating countries. Informed consent was obtained prior to participation for all enrolled women.

### Data analysis

Due to differing characteristics in setting and study procedures, the relationship of postpartum blood loss and hemoglobin outcomes was assessed separately for the Pakistan community-based PPH prevention study and the two multisite hospital-based PPH treatment studies. We excluded the following women from analysis: did not have information on measured postpartum blood loss (n = 44, all in Pakistan study); did not have information on pre- and post-delivery hemoglobin (n = 14 in Pakistan study, n = 7 in multisite studies); had post-delivery hemoglobin measured <12 hours after delivery (n = 412, all in the multisite studies); received a blood transfusion (n = 85, all in the multisite studies). We excluded women with post-delivery hemoglobin measured <12 hours after delivery because hemoglobin levels will not reflect blood loss if measured too soon after the event [22]. These exclusions account for differences in numbers reported here and in the primary publications of these studies [9, 10]. In the multisite studies, pre-and post-delivery hemoglobin was only measured for women diagnosed with PPH, thus no data were available on hemoglobin drop for women with <500ml blood loss. For both pre- and post-delivery hemoglobin measurements, hemoglobin values were adjusted when measurements were taken among women from higher elevations, as recommended by the WHO [23]. Thus, we subtracted 0.5 g/L from hemoglobin measurements taken in Pakistan prevention study (done in Chitral, elevation 1493 meters) and we subtracted 1.5 g/dL from measurements taken at the Ecuador site of the multisite treatments (done in Quito, elevation 2850 meters).

The trends in change in hemoglobin and in postpartum hemoglobin measurement with different levels of blood loss were assessed by plotting the moving average and 95% confidence intervals (CIs) of hemoglobin drop by 100 ml intervals of blood loss. A moving average was used due to small numbers of women in the higher intervals of blood loss, particularly in the Pakistan study. The moving average of hemoglobin drop was calculated for each 100 ml interval by averaging the hemoglobin drops measured within the interval and the drops measured in the preceding and subsequent 100 ml intervals.

We calculated the Spearman’s rank correlation coefficient (*r*_*s*_) for the relationship of measured blood loss and hemoglobin drop and of blood loss and postpartum hemoglobin. This non-parametric method was used because the continuous variable of blood loss was not normally distributed. We used the categories described by Hinkle et al to characterize the correlation based on the value of *r*_*s*_ (i.e. 0 to -0.3: negligible correlation, -0.3 to -0.5 low correlation, -0.5 to -0.7 moderate correlation, -0.7 to -0.9 high correlation, -0.8 to -1.0 very high correlation) [24]. Since there was variability in the two studies regarding when pre-delivery hemoglobin and post-delivery hemoglobin were measured, we performed a sensitivity analysis to assess if the strength of correlation differed by timing of hemoglobin was measurement. For the Pakistan prevention study, this sensitivity analysis included comparing correlations according to whether pre-delivery hemoglobin was measured <1 week, 1–4 weeks, or >4 weeks before delivery, and according to whether post-delivery hemoglobin was measured ≤3 days or 4–5 days after delivery. For the multisite treatment studies, hemoglobin was measured during labor for all participants so the sensitivity analysis for post-delivery hemoglobin compared Spearman correlation coefficients according to whether post-delivery hemoglobin was measured <1 day, 1–3 days, or >3 days after delivery. We also performed a second sensitivity analysis to determine if the strength of correlation differed by pre-existing anemia (defined as pre-delivery hemoglobin <10 g/dL).

Categorical analysis was done to determine how often women with bleeding that met the common blood loss definitions of PPH (≥500 ml, ≥1000 ml) experienced the outcome of hemoglobin drop ≥2 g/dL (a threshold commonly used to indicate a clinically important drop in hemoglobin) [14], as well as thresholds for moderate postpartum anemia (postpartum hemoglobin <10 g/dL) and severe postpartum anemia (postpartum hemoglobin <7 g/dL) [23]. We calculated proportions and 95% CIs of women with hemoglobin drop ≥2 g/dL and of women with postpartum hemoglobin that fell below the thresholds for moderate and severe postpartum anemia and stratified by blood loss <500 ml, 500–999 ml, and ≥1000 ml for women in the Pakistan prevention study and by blood loss 500–999 ml and ≥1000 ml for women in the multisite treatment studies. The 95% CIs were calculated with the Z test for proportions using the prtest function in Stata. All analyses were performed using Stata 12 (StataCorp. 2011. *Stata Statistical Software*: *Release 12*. College Station, TX).

## Results and discussion

Differing characteristics of the study populations for the Pakistan PPH prevention study and the multisite PPH treatment studies are depicted in **Table 1**. Women in the Pakistan prevention study had varying amounts of recorded blood loss (0–1890 ml), whereas the multisite treatment study only included women with clinical diagnosis of PPH and/or measured blood loss ≥700 ml (range: 450–2300 ml). Compared to the Pakistan prevention study, women in the multisite treatment studies had lower mean pre-delivery hemoglobin (12.3 g/dL vs. 11.6 g/dL), a higher proportion of women with pre-delivery <11 g/dL (19.6% vs. 33.2%) higher mean blood loss (352 ml vs. 1007 ml), larger drops in pre- to post-delivery hemoglobin (-1.2 g/dL vs. -2.2 g/dL), and more frequent use of interventions such as IV fluids (0% vs. 100%).

**Table 1:** Participant characteristics in the Pakistan postpartum hemorrhage (PPH) prevention study and the multisite PPH treatment studies*.

	Pakistan PPH prevention study, N = 1058	Multisite PPH treatment studies, N = 1283
**Place of delivery**		
Home	1058 (100%)	0
Hospital	0	1283 (100%)
**Age (years)**		
<25	263 (24.9%)	712 (55.5%)
25–34	697 (65.9%)	487 (38.0%)
>35	98 (9.3%)	84 (6.5%)
**Parity**		
Nulliparous	211 (19.9%)	727 (56.7%)
1–2	479 (45.3%)	468 (36.5%)
≥3	368 (34.8%)	88 (6.9%)
**Pre-delivery Hb**		
Mean (SD)	12.3 (1.6)	11.6 (1.4)
<11.0 g/dL	207 (19.6%)	426 (33.2%)
**Use of intravenous fluids during labor and/or postpartum**	0	1283 (100%)
**Use of uterotonics**		
During 3^rd^ stage of labor	507 (48.0%)	747 (58.2%)
After PPH diagnosis	14 (1.3%)	1283 (100%)
**Blood loss**		
Mean (SD)	352 (246.1)	1007 (261)
<500 ml	853 (80.8%)	3 (0.2%)
500–999 ml	175 (16.6%)	725 (56.4%)
≥1000 ml	28 (2.7%)	555 (43.3%)
**Change in pre- to post-delivery Hb** **Mean (SD)**	-1.2 (1.3)	-2.2 (1.7)
**Post-delivery Hb** **Mean (SD)**	11.1 (1.6)	9.3 (1.5)
**Time post-delivery Hb measured (days after delivery), median (range)**	4.0 (2.0, 6.0)	1.1 (0.5, 7.9)

*Results are presented as N (%) except where otherwise stated

### Mean drop in pre- to post-delivery hemoglobin and mean postpartum hemoglobin

**Fig 1:**
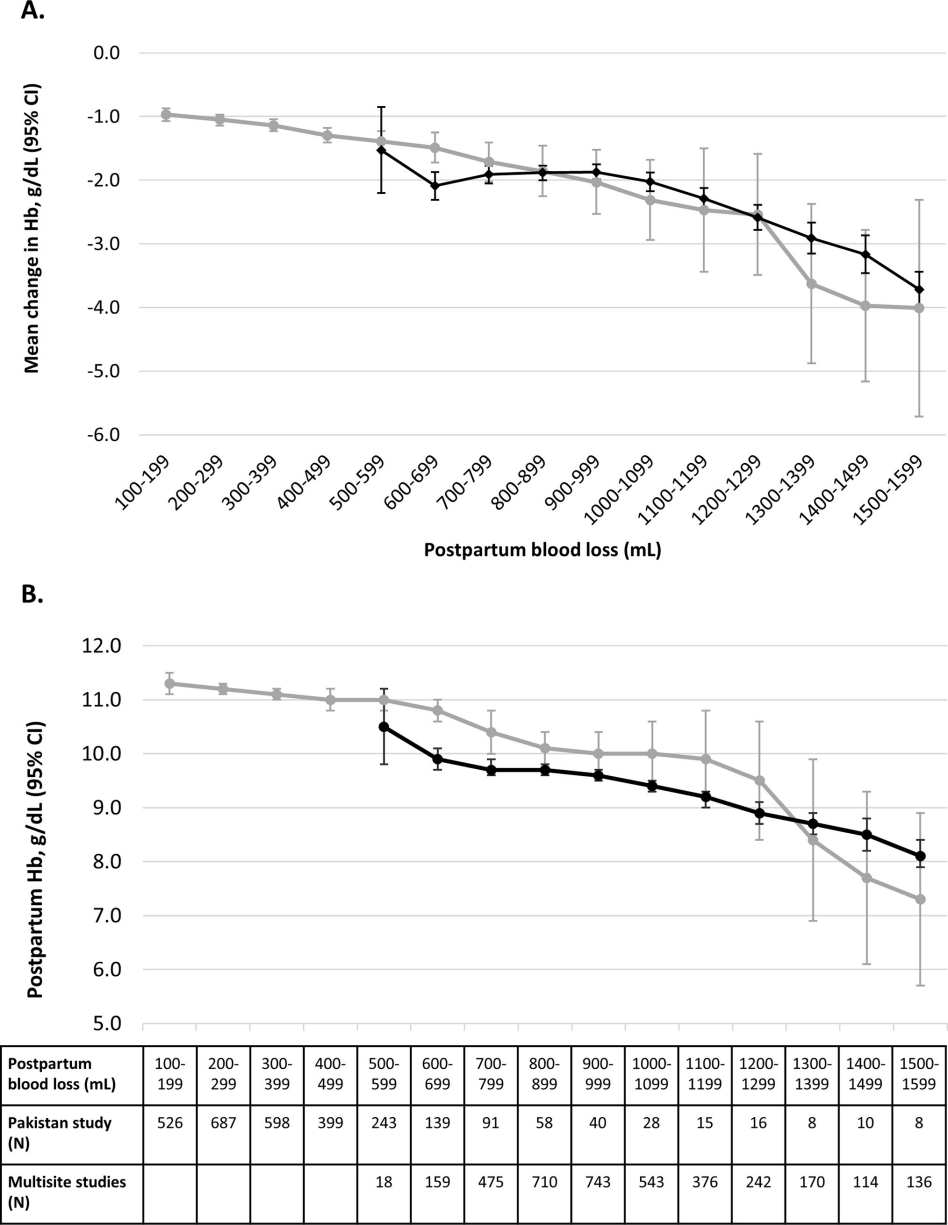
Moving average* of change in pre- to post-delivery hemoglobin (Fig 1A) and postpartum hemoglobin (Fig 1B) by postpartum blood loss (ml) in the Pakistan PPH prevention study (N = 1058) and the multisite PPH treatment studies (N = 1283). *Data from the Pakistan prevention study are depicted in grey and data from the multisite treatment studies are depicted in black. The circles represent the moving average of change in hemoglobin or postpartum hemoglobin at each blood loss interval and the brackets represent the 95% confidence intervals (CIs) of the moving average. The moving average for each 100ml interval reflects the mean hemoglobin drop or mean postpartum hemoglobin measured within the interval and the preceding and subsequent 100 ml intervals. The number of women included in calculation of the moving average at each blood loss interval is indicated in the data table.

The moving average of change in hemoglobin gradually dropped over 100 ml intervals of blood loss (**Fig 1A)**. Mean hemoglobin drops were above the clinically important value of ≥2 g/dL after blood loss exceeded 900 ml in the Pakistan prevention trial and 1100 ml in the multisite treatment studies. Mean postpartum hemoglobin fell below the marker of moderate anemia (10.0 g/dL) when blood loss exceeded 1100 ml in the Pakistan prevention trial and 600 ml in the multisite treatment trials. Mean postpartum hemoglobin levels did not fall below the marker of severe anemia (7.0 g/dL) in any blood loss categories below 1600 ml in the Pakistan prevention study or the multisite treatments trials (**Fig 1B**).

Blood loss was significantly correlated with change in hemoglobin and with postpartum hemoglobin in the Pakistan prevention trial and multisite treatment studies (p<0.001, **Table 2**). However, the Spearman correlation coefficients (***r***_***s***_) for relationship between blood loss and change in pre- to post-delivery hemoglobin were -0.220 in Pakistan trial and -0.271 in the multisite trials, indicating a negligible correlation between the two variables [24]. Similarly, the correlation between blood loss and postpartum hemoglobin was negligible in the Pakistan prevention study (***r***_***s***_ = -0.220) and was low in the multisite treatment studies (***r***_***s***_ = -0.316). Sensitivity analysis around time of pre-delivery and post-delivery hemoglobin measurements shows that the strength of correlation differed slightly depending on when these measurements were taken; however, the correlation coefficients never exceeded values that would indicate anything higher than “low” strength of correlation of blood loss with hemoglobin outcomes (**Table A in S1 Appendix**). The second sensitivity analysis also showed that correlation remained in the negligible to low categories regardless of pre-existing anemia (i.e. pre-delivery hemoglobin <10 g/dL, see **Table B in S1 Appendix**).

**Table 2:** Correlation of postpartum blood loss with change in pre- to post-delivery hemoglobin and postpartum hemoglobin.

	Spearman’s correlation coefficient	
	*r*_*s*_	p value
**PAKISTAN PREVENTION STUDY, n = 1058**		
Change in pre- to post-delivery Hb	-0.220	<0.001
Post-delivery Hb	-0.220	<0.001
**MULTISITE TREATMENT TRIALS, n = 1283**		
Change in pre- to post-delivery Hb	-0.271	<0.001
Post-delivery Hb	-0.316	<0.001

### Pre- to post-delivery hemoglobin drop ≥2 g/dL

Among 855 women with blood loss <500 ml in the Pakistan prevention study, less than a quarter of women had hemoglobin drop ≥2 g/dL or moderate postpartum anemia, and rates of sever postpartum anemia were less than 1% (**Table 3**). Among women with blood loss 500–999 ml both in the Pakistan and multisite treatment trials, a minority had a hemoglobin drop ≥2 g/dL (55/175 [31%] in Pakistan study and 302/725 [42%] in multisite trials), the same or slightly larger proportion of women had postpartum hemoglobin below the threshold of moderate anemia (55/175 [31%] in Pakistan study and 390/725 [54%] in multisite trials), and severe anemia was uncommon (2/175 [1%] in Pakistan study and 25/725 [3%] in multisite trials). In all studies, the majority of women with blood loss >1000 ml had hemoglobin drop ≥2 g/dL (19/28 [68%] in Pakistan study and 347/554 [63%] in multisite trials) and postpartum hemoglobin below the threshold of moderate postpartum anemia (20/28 [71%] in Pakistan study and 416/554 [75%] in multisite trials), though the rate of severe postpartum anemia was still fairly low (3/28 [14%] in Pakistan study and 57/554 [10%] in multisite trials).

**Table 3:** Association of common threshold definitions of postpartum hemorrhage with a clinically important drop in pre- to post-delivery hemoglobin ≥2 g/dL, moderate postpartum anemia (i.e. hemoglobin <10 g/dL), and severe postpartum anemia (i.e. hemoglobin <7 g/dL).

	Total, N	Hemoglobin drop ≥2 g/dL		Moderate postpartum anemia or worse (Hb<10 g/dL)		Severe postpartum anemia (Hb<7 g/dL)	
		N	Proportion (95% CI)	N	Proportion (95% CI)	N	Proportion (95% CI)
**Pakistan prevention study**							
**Total**	**1058**	**199**	**19% (17%–21%)**	**252**	**24% (21%–27%)**	**10**	**1% (17%–21%)**
Blood loss							
<500 ml	855	125	15% (13%–17%)	177	21% (18%–24%)	4	<1% (<1%–1%)
500–999 ml	175	55	31% (24%–38%)	55	31% (24%–38%)	2	1% (<1%–2%)
≥ 1000 ml	28	19	68% (51%–85%)	20	71% (54%–88%)	3	14% (1%–27%)
**Multisite treatment trials**							
**Total**	**1280**	**649**	**51% (48%–54%)**	**806**	**63% (60%–66%)**	**82**	**6% (5%–7%)**
Blood loss							
500–999 ml^1^	725	302	42% (38%–46%)	390	54% (50%–58%)	25	3% (2%–4%)
≥ 1000 ml	554	347	63% (59%–67%)	416	75% (70%–78%)	57	10% (8%–12%)

^1^For multisite treatment study, the majority of women in this category bled between 700–999 ml.

Postpartum blood loss and change in pre- to post-delivery hemoglobin are commonly regarded as interchangeable, with the assumption that an estimation of blood loss can help approximate the change in hemoglobin and vice versa. For example, researchers have used change in hemoglobin to validate visual or indirect methods of blood loss estimation [25, 26]. However, the present findings of data from two distinct settings (community-based and hospital-based births) indicate that postpartum blood loss is a poor predictor of pre- to post-delivery change in hemoglobin and postpartum hemoglobin. Though the relationship between blood loss and hemoglobin outcomes was statistically significant (e.g. the change of hemoglobin increased as the amount of blood loss increased), the correlation coefficients noted in this study ranged from -0.220 to -0.316; these values indicate that the strength of the relationship between blood loss and hemoglobin outcomes is negligible to low^23^ (as 0 denotes a weak correlation and coefficients that approach -1 or 1 denote a strong correlation). The findings of low degree of correlation between blood loss and change in hemoglobin corroborate results documented by others.[14, 15, 25]

Overall, our findings suggest a minor role of the exact quantity of blood loss in predicting a woman’s postpartum condition. It is striking that this finding is consistent in a community setting in Pakistan, where minimal interventions are available, and in hospital-based settings in 5 countries, where an ample array of interventions (including administration of intravenous fluids) are widely used. As for why blood loss and hemoglobin measurement were so poorly correlated in these obstetric populations, a conclusion proposed by authors of another study that documented similar findings is that the physiological adaptions of pregnancy provide protection from large drops in hemoglobin even following above-average blood loss (500-1200ml). This could be related to the overall increase in plasma volume that occurs during pregnancy, itself considered a protective adaption from the anticipated acute blood loss at the time of delivery.[22, 27]

Perhaps most importantly, our findings suggest that the 500 ml threshold is a metric of limited utility for evaluating outcomes associated with PPH. A clinically important drop in hemoglobin of ≥2 g/dL did not occur with frequency until blood loss exceeded 900–1100 ml. Further, among women with blood loss that exceeded 500 ml but was below 1000 ml, only 31–42% experienced hemoglobin drop ≥2 g/dL and 31–54% postpartum hemoglobin below the threshold of moderate anemia. The fact that more women with blood loss 500–999 ml in the multisite treatment trials had postpartum hemoglobin below the marker of moderate anemia compared to women with the same among of blood loss in the Pakistan prevention trial may be explained by the fact that women in the multisite treatment trials had a lower mean pre-delivery hemoglobin; in this respect, change in hemoglobin is likely a better indicator of the effect of blood loss. Notably, just 1–3% of women with blood loss 500–999 ml had postpartum hemoglobin below the threshold of “severe” anemia, when blood transfusion is commonly indicated [28]. Ultimately, our data suggest that the 500 ml threshold (which represents just 10% of total blood volume in the average full-term pregnant woman) may not be an appropriate indicator of a negative outcome, as many women tolerate this amount of blood loss without complication, though this may differ according to the presence of pre-delivery anemia or other underlying health conditions.[17, 29] In terms of blood-loss based definitions of PPH, the 1000 ml threshold appears more useful, as this is when clinically important drops in hemoglobin occurred with greater frequency and when the majority of women had postpartum hemoglobin values below the marker of moderate anemia.

When considering the potential implications of this analysis beyond research and for clinical practice, it is important to note that our analysis focused on the endpoint of blood loss, and in practice, one cannot know if blood loss that exceeds 500 ml will mark the end of bleeding (in which case minimal intervention is likely necessary) or an earlier point in the course of bleeding (in which case more rigorous intervention is likely necessary). Thinking along these lines, we believe that these results support the recommendation made in a WHO 1996 statement, which states that the 500ml threshold “*should be considered an alert line; the action line is then reached when vital functions of the woman are endangered*. *In healthy women this usually only occurs after blood loss >1000ml*.*”* [30] The results also support the ACOG recommendations which state that “blood loss of 500–999 ml alone should trigger increased supervision…” but that the criteria for PPH has not been met until blood loss exceeds 1000ml [11]. However, in remote areas where access to emergency obstetric care is limited, 500ml may be the appropriate threshold for administering first-line interventions such as uterotonics.

Our findings also support the recommendation that future PPH definitions move beyond blood loss [8]. The pitfalls associated with estimating blood loss are well-documented [22, 31] and the exact amount of blood loss is not known following most deliveries. Close attention to the woman’s overall condition during the postpartum period, including regular measurement of vital signs, is essential yet not always regularly practiced in busy settings with limited resources. Along these lines, research is underway to better define the role of shock index (ratio of systolic blood pressure to pulse rate) as an additional indicator of PPH [32–35].

This analysis has some limitations. It includes secondary analyses of data obtained from three studies that had aims and objectives distinct from those of this analysis. However, all efforts were made in those studies to measure blood loss and pre- and post-delivery hemoglobin systematically. The included studies were heterogenous regarding the methods of objective blood loss measurement and timing of pre- and post-delivery hemoglobin assessment. However, the blood loss measurement methods used in all three studies have been used in previous studies and shown to be useful and accurate, particularly in comparison to visual assessment of blood loss [36, 37]. There was some variability in the timing of measurement of pre-delivery hemoglobin in the Pakistan study and in the timing of post-delivery hemoglobin in both the Pakistan and multisite studies, which could have impacted observed correlations; however, our sensitivity analysis revealed our findings showing a low correlation were robust to the timing of these measurements. The findings around low strength of correlation were also robust to presence of pre-delivery anemia. The measurements of post-delivery hemoglobin may have been impacted by postpartum diuresis or dehydration or, alternatively, by hydration with IV fluids; these factors could have led to a potential underestimation or overestimation of hemoglobin drop pre- to post-delivery. Additionally, pre- and post-delivery hemoglobin levels were estimated using the Hemocue device, which has been shown to give slightly higher readings compared to automated laboratory methods; this may have led to some underestimations of postpartum hemoglobin and rates of postpartum anemia, but it should not have impacted our analysis of fall in hemoglobin [38, 39]. Nevertheless, our conclusions are strengthened by the fact that we document consistent results in different delivery settings (including community-based and hospital-based settings) that enhance the generalizability of our findings.

## Conclusions

Our results suggest that the PPH definition of ≥500 ml blood loss is poorly correlated with PPH-related morbidity as measured by fall in hemoglobin and postpartum anemia. These findings call into question the meaningfulness of this threshold as an endpoint in research to assess the effectiveness of PPH interventions. Additional studies and potential meta-analyses may shed further light on the relationship of postpartum blood loss and hemoglobin outcomes. Our results suggest that future studies assessing PPH interventions should attach less importance to endpoints like ≥500 mL and aim for including more meaningful outcomes like severe PPH. This approach may improve identification of interventions that are most likely to result in lower maternal mortality and morbidity.

## Supporting information

S1 Appendix Sensitivity analyses considering timing of pre- and post-delivery hemoglobin measurements and existence of pre-delivery anemia when assessing the association of postpartum blood loss with hemoglobin markers. (DOCX)
