# Supplementary material for: How well do postpartum blood loss and common definitions of postpartum hemorrhage correlate with postpartum anemia and fall in hemoglobin?
Source: PLoS One. 2019 Aug 22;14(8):e0221216. doi: 10.1371/journal.pone.0221216 (PMC6705817; doi:10.1371/journal.pone.0221216)
Supplement: S1 Appendix — (DOCX) [file pone.0221216.s001.docx]

How well do postpartum blood loss and common definitions of postpartum hemorrhage correlate with postpartum anemia and fall in hemoglobin?

Holly A. Anger^1^*, Jill Durocher^1^, Rasha Dabash^1^, Beverly Winikoff^1^

**S1 Appendix: Sensitivity analyses considering timing of pre- and post-delivery hemoglobin measurements and existence of pre-delivery anemia when assessing the association of postpartum blood loss with hemoglobin markers.**

**Table A:** Spearman’s correlation coefficient (***r_s_*** ) of postpartum blood loss with change in pre- to post-delivery hemoglobin and postpartum hemoglobin with sensitivity analysis of timing of pre- and post-delivery hemoglobin measurement.

| Pre-delivery Hb – time measured before delivery | Post Hb – time measure after delivery | | | |
| --- | --- | --- | --- | --- |
|  | <=24 hrs | 1-3 days | 4-5 days | Total |
|  | *r_s_* (N) | *r_s_* (N) | *r_s_* (N) | *r_s_* (N) |
| Pakistan prevention study |  |  |  |  |
| Correlation: Blood loss and Hb change |  |  |  |  |
| <=1 week | NA | -0.25 (n=64) | -0.22 (n=186) | -0.23 (n=250) |
| 1-4 weeks | NA | -0.36 (n=55) | -0.23 (n=248) | -0.25 (n=303) |
| >4 weeks | NA | -0.16 (n=111) | -0.20 (n=392) | -0.19 (n=504) |
| Total | NA | -0.24 (n=230) | -0.21 (n=826) | -0.22 (n=1057) |
| Correlation: Blood loss and postpartum Hb |  |  |  |  |
| <=1 week | NA | -0.37 (n=64) | -0.24 (n=186) | -0.27 (n=250) |
| 1-4 weeks | NA | -0.36 (n=55) | -0.17 (n=248) | -0.22 (n=303) |
| >4 weeks | NA | -0.21 (n=111) | -0.20 (n=392) | -0.20 (n=504) |
| Total | NA | -0.30 (n=230) | -0.20 (n=826) | -0.22 (n=1057) |
| Multisite treatment studies* |  |  |  |  |
| Correlation: Blood loss and Hb change | -0.46 (n=560) | -0.23 (n=488) | -0.10 (n=235) | -0.27 (n=1283) |
| Correlation: Blood loss and postpartum Hb | -0.37 (n=560) | -0.36 (n=488) | -0.17 (n=235) | -0.31 (n=1283) |

*In multisite treatment studies, pre-delivery Hb was measured for all women during labor, thus sensitivity analysis was not needed for that variable.

**Table B:** Spearman’s correlation coefficient of postpartum blood loss with change in pre- to post-delivery hemoglobin and postpartum hemoglobin with sensitivity analysis of pre-delivery anemia

|  | Pre-delivery Hb ≥10 g/dL | | Pre-delivery Hb <10 g/dL | |
| --- | --- | --- | --- | --- |
|  | ***r_s_*** | **p value** | ***r_s_*** | **p value** |
| Pakistan prevention study | N=976 |  | N=82 |  |
| Correlation: Blood loss and Hb change | -0.23 | <0.001 | -0.20 | 0.07 |
| Correlation: Blood loss and postpartum Hb | -0.22 | <0.001 | -0.22 | 0.04 |
| Multisite treatment studies* | N=1161 |  | N=122 |  |
| Correlation: Blood loss and Hb change | -0.28 | <0.001 | -0.26 | 0.004 |
| Correlation: Blood loss and postpartum Hb | -0.34 | <0.001 | -0.14 | 0.139 |
